# TLR2 Ligand Pam3CSK4 Regulates MMP-2/9 Expression by MAPK/NF-κB Signaling Pathways in Primary Brain Microvascular Endothelial Cells

**DOI:** 10.1007/s11064-018-2607-7

**Published:** 2018-08-07

**Authors:** Hongyan Zhu, Rongrong Dai, Youquan Zhou, Hao Fu, Qiang Meng

**Affiliations:** 1 Faculty of Environmental Science and Engineering, Kunming University of Science and Technology, Kunming, 650093 Yunnan Province China; 2 Department of Clinical Laboratory, The Affiliated Hospital of Kunming University of Science and Technology, Kunming, 650031 Yunnan Province China; 3 Department of Clinical Laboratory, The First People’s Hospital of Yunnan Province, Kunming, 650031 Yunnan Province China; 4 Medical Faculty of Kunming University of Science and Technology, Kunming, 650093 Yunnan Province China; 5 Department of Clinical Laboratory, The First Affiliated Hospital of Kunming Medical University, Kunming, 650034 Yunnan Province China; 6 Department of Clinical Laboratory, The Third Affiliated Hospital of Kunming Medical University, Kunming, 650118 Yunnan Province China; 7 Department of Neurology, The First People’s Hospital of Yunnan Province, Kunming, 650031 Yunnan Province China

**Keywords:** Toll-like receptor-2, Matrix metalloproteinase, Pam3CSK4, Mitogen-activated protein kinases, Brain microvascular endothelial cells

## Abstract

Blood–brain barrier (BBB) destruction is associated with a variety of neurological diseases. Brain microvascular endothelial cells (BMECs) are the key constituent of BBB. Both matrix metalloproteinases-2/9 (MMP-2/9) and toll-like receptor-2 (TLR2) are coexpressed in BMECs and have been shown to play important roles in BBB breakdown. It is unknown whether TLR2 can regulate MMP-2/9 in BMECs. In this study, Pam3CSK4 was used to activate TLR2, and the expression of MMP-2/9 and tight junctions (TJs) in BBB was measured by quantitative real-time PCR and western blotting. Phosphoproteins were determined by western blotting. The inhibitors of mitogen-activated protein kinases (MAPKs) and NF-κB were used to identify the signaling pathways by which TLR2 regulates the expression of MMP-2/9 in BMECs. This study showed that Pam3CSK4 upregulated the mRNA and protein expression of MMP-9 and downregulated MMP-2 and TJ expression in BMECs simultaneously. Pam3CSK4 also induced the phosphorylation of MAPKs and NF-κB signaling pathways in BMECs. MMP-9 expression was found to decrease by pretreatment with inhibitors of ERK1/2 and JNK but not p38. However, the mRNA and protein expression of MMP-2 and MMP-9 increased after addition of a NF-κB inhibitor. Our results indicated that Pam3CSK4 was able to upregulate MMP-9 expression through ERK1/2 and JNK signaling pathways, but the NF-κB signaling pathway negatively regulated the effect of TLR2 on MMP-2 and MMP-9 expression in BMECs. The finding provides novel insight into the molecular mechanism of MMP-2/9 expression in BMECs.

## Introduction

Toll-like receptors (TLRs) are transmembrane pattern recognition receptors (PRRs) that are involved not only in systemic bacterial infection but also in cerebral injury [1]. TLRs are expressed in mammalian innate immune cells and non-immune cells, such as epithelial and endothelial cells [1]. Almost all cell types in the central nervous system (CNS), including microglia, neurons, astrocytes [2], and endothelial cells [3], express TLRs. Thirteen murine and 10 human TLRs are currently known [4, 5]. TLR2 is one TLR that is expressed on the cell surface [2]. A previous study showed that the mRNA expression of TLR2 was upregulated in a mouse model of cerebral ischemia [6]. Compared with that in wild-type mice, the infarct size of TLR2-deficient mice was reduced following cerebral focal ischemia injury [6].

The blood**–**brain barrier (BBB) plays a pivotal role in maintaining the homeostasis of the CNS microenvironment. BBB is formed by brain microvascular endothelial cells (BMECs) linked by tight junctions (TJs) and adherens junctions (AJs) [7]. TJs and AJs between endothelial cells maintain the integrity of the BBB [3]. BBB disruption is related to a series of CNS diseases, such as multiple sclerosis [7], hypoxia, and ischemia [8].

Matrix metalloproteinases (MMPs) are a family of zinc-dependent enzymes that disrupt the BBB integrity by degrading TJs of endothelial cells (ECs) [9]. MMP-2/9 degrade the main constituents of the basal lamina, including type IV collagen, lamin, and fibronectin, around the cerebral blood vessels [9]. Recent reports have shown that both MMP-2/9 and TLR2 [3] are expressed in BMECs. TLR2 can activate the mitogen-activated protein kinase (MAPK) pathway [3]. Other studies have shown MMPs are regulated by the MAPK signaling pathway [10]. However, the molecular mechanism has not been studied before. We speculate that TLR2 activation may induce MMP-2/9 expression by MAPK and NF-κB signaling pathways in BMECs, resulting in BBB disruption. Therefore, we used TLR2 synthetic analogue ligand Pam_3_Cys–Ser–Lys_4_ (Pam3CSK4) to activate TLR2 and explore whether and how Pam3CSK4 regulates MMP-2/9 expression in BMECs.

## Materials and Methods

### Reagents

TLR2 agonist Pam3CSK4 was purchased from InvivoGen (San Diego, CA, USA). U0126 (ERK1/2 inhibitor) was purchased from Cell Signaling Technology (Beverly, MA, USA). SB203580 (p38 MAPK inhibitor), SP600125 (JNK inhibitor), and BAY11-7082 (NF-κB inhibitor) were purchased from Calbiochem (San Diego, CA, USA).

For western blot analysis, anti-TLR2 (catalog ab108998; 1:1000), anti-MMP-9 (catalog ab76003; 1:1000), anti-MMP-2 (catalog ab37150; 1:1000), and anti-occludin (catalog ab167161; 1:1000) were purchased from Abcam (Shanghai, China). Anti-claudin 5 (catalog #ABT45; 1:1000) and anti-collagen IV (catalog ab6586; 1:2000) were purchased from Merck Millipore (Billerica, MA, USA) and Abcam (Shanghai, China), respectively. Anti-ZO-1 (catalog 61-7300; 1:4000) was purchased from Invitrogen (Carlsbad, CA, USA). β-actin was from Proteintech (Rosemont, IL, USA). Antibodies against phospho-ERK1/2 (catalog #4377), pJNK (catalog #4668), pP38 MAPK (catalog #4511), and pNF-κB p65 (catalog #3033) were purchased from Cell Signaling Technology.

### Primary Brain Microvascular Endothelial Cell (BMEC) Culture

All experiments were performed in accordance with the National Institutes of Health (USA) Guide for the Care and Use of Laboratory Animals and approved by the Animal Care Committee of Kunming University of Science and Technology, China. BMECs were cultured from Sprague–Dawley neonatal rat cerebral cortices as published previously [11]. Endothelial cells were cultured in DMEM/high glucose with 20% fetal bovine serum (FBS) for 24 h and selected with 4 µg/mL puromycin (Amresco, Ohio, USA). BMECs were identified by immunofluorescence staining with von Willebrand factor (vWF, 1:50, Proteintech, Rosemont, IL, USA).

### Quantitative Real-Time Polymerase Chain Reaction

Total RNA was isolated from BMECs using Eastep™ Total RNA Extraction Kit (Promega, Shanghai, China). The quality and quantity of isolated RNA were measured by NanoDrop2000 (Thermo Fisher Scientific, Waltham, MA, USA). First-strand cDNA was synthesized with the GoScript™ Reverse Transcription System (Promega) according to the manufacturer’s protocol. Quantitative real-time polymerase chain reaction (qRT-PCR) was conducted with 1 uL cDNA products using SYBR® Premix Ex TaqTM II (TliRNaseH Plus, Takara, Dalian, China) on a Roche LightCycler 480.

*Primers*. β-actin, MMP-2, and MMP-9 rat primers were designed as follows:

β-actin (forward: 5′-GGAGATTACTGCCCTGGCTCCTA-3′, reverse: 5′-GACTCATCGTACTCCTGCTTGCTG-3′);

MMP-2 (forward: 5′-ACCTTGACCAGAACACCATCGAG-3′, reverse: 5′-CAGGGTCCAGGTCAGGTGTGTA-3′);

MMP-9 (forward: 5′-CATGCGCTGGGCTTAGATCA-3′, reverse: 5′-GAGGCCTTGGGTCAGGTTTAGAG-3′). The PCR conditions were as follows: one cycle of initial denaturation (95 °C for 30 s), amplification cycles (40 cycles of 95 °C for 5 s, 55 °C for 30 s, and 72 °C for 30 s), and one cycle of amplification curve analysis (95 °C for 5 s, 60 °C for 60 s, and 95 °C). Each reaction was repeated three times. The comparative mRNA expression level was expressed as 2^−ΔΔCt^.

### Western Blot Analysis

Cell lysates were harvested with RIPA lysis buffer (Beyotime Biotechnology, Shanghai, China) containing Protease Inhibitor Cocktail (Calbiochem, San Diego, CA, USA) and PhosSTOP (Roche Applied Science, Rockford, IL, USA). Protein concentrations in supernatant were detected by the bicinchoninic acid (BCA) assay (Beyotime Biotechnology). Proteins were separated by western blot and then transferred to polyvinylidene difluoride membranes (Merck Millipore), which were then blocked with Tris-buffered saline (TBS; Sangon Biotech, Shanghai, China) containing 0.1% Tween 20 with 5% (w/v) non-fat milk. Membranes were incubated overnight at 4 °C with the primary antibody and then incubated for 2 h at room temperature with horseradish peroxidase (HRP)-conjugated secondary antibody (1:5000, Proteintech). Antibody dilution ratios were as follows: TLR2 antibody (1:1000), MMP-9 antibody (1:1000), MMP-2 antibody (1:1000), occludin antibody (1:1000), claudin 5 antibody (1:1000), collagen IV antibody (1:2000), ZO-1 antibody (1:4000), β-actin antibody (1:5000), and p-ERK1/2, p-JNK, p-p38, and p-NF-κB p65 antibody (1:1000). Bands were visualized with enhanced chemiluminescence (Proteintech) and photographed using a membrane imaging system (Bio-Rad, Hercules, CA, USA). Band intensity was semi-quantitatively measured by ImageJ software (NIH, Bethesda, MD, USA).

### Statistical Analysis

The results were expressed as means ± standard error (SE). Statistical analyses were performed using one-way analysis of variance (ANOVA) followed by the least significant difference test. *p* ≤ 0.05 was considered statistically significant.

## Results

### Pam3CSK4 Upregulated MMP-9 Expression But Downregulated MMP-2 Expression in BMECs

**Fig. 1:**
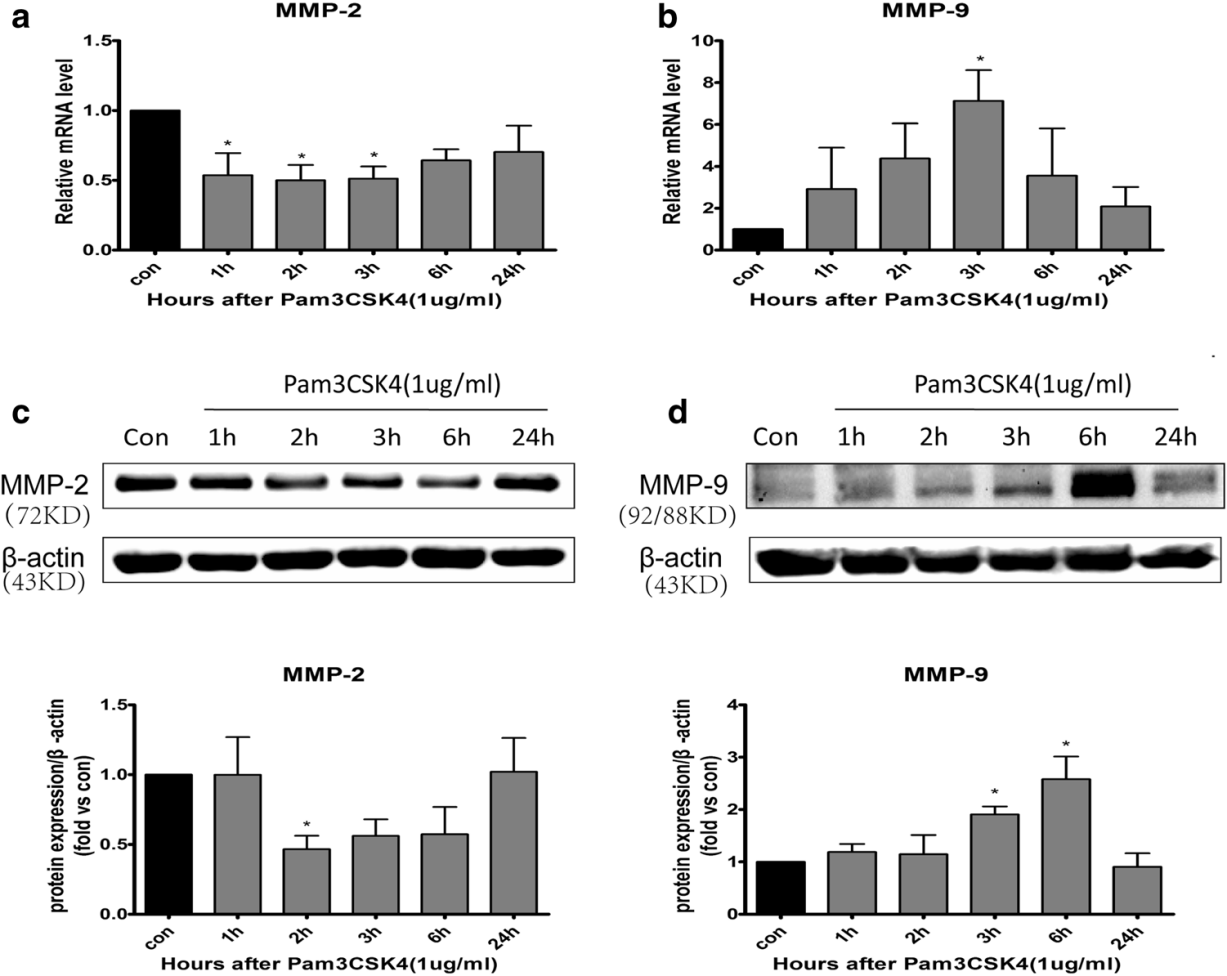
The mRNA and protein expression levels of MMP-2 and MMP-9 in BMECs stimulated with Pam3CSK4. BMECs were stimulated with Pam3CSK4 (1 µg/mL) for 1, 2, 3, 6, and 24 h. The mRNA expression levels of MMP-2 (**a**), MMP-9 (**b**), and β-actin were analyzed by qRT-PCR. Cells were collected for detection of MMP-2 (**c**) and MMP-9 (**d**) protein expression by western blot, and protein levels were quantified by ImageJ software and normalized with β-actin protein levels. *p < 0.05, **p < 0.001 as compared with control group, in which cells were treated with PBS

It has been reported that BMECs can express both MMPs [12] and TLR2 [3]. To investigate the effect of TLR2 activation on MMP-2/9 in BMECs, we treated cells with 1 µg/mL Pam3CSK4 for 1, 2, 3, 6, and 24 h. The mRNA and protein expression of MMP-9 increased at 3 and 6 h, respectively (*p* < 0.05, Fig. 1b, d). The mRNA level of MMP-2 significantly decreased at 1, 2, and 3 h (*p* < 0.05, Fig. 1a). However, MMP-2 protein levels decreased at 2 h (*p* < 0.05, Fig. 1c).

### Pam3CSK4 Downregulated TJ Expression in BMECs

**Fig. 2:**
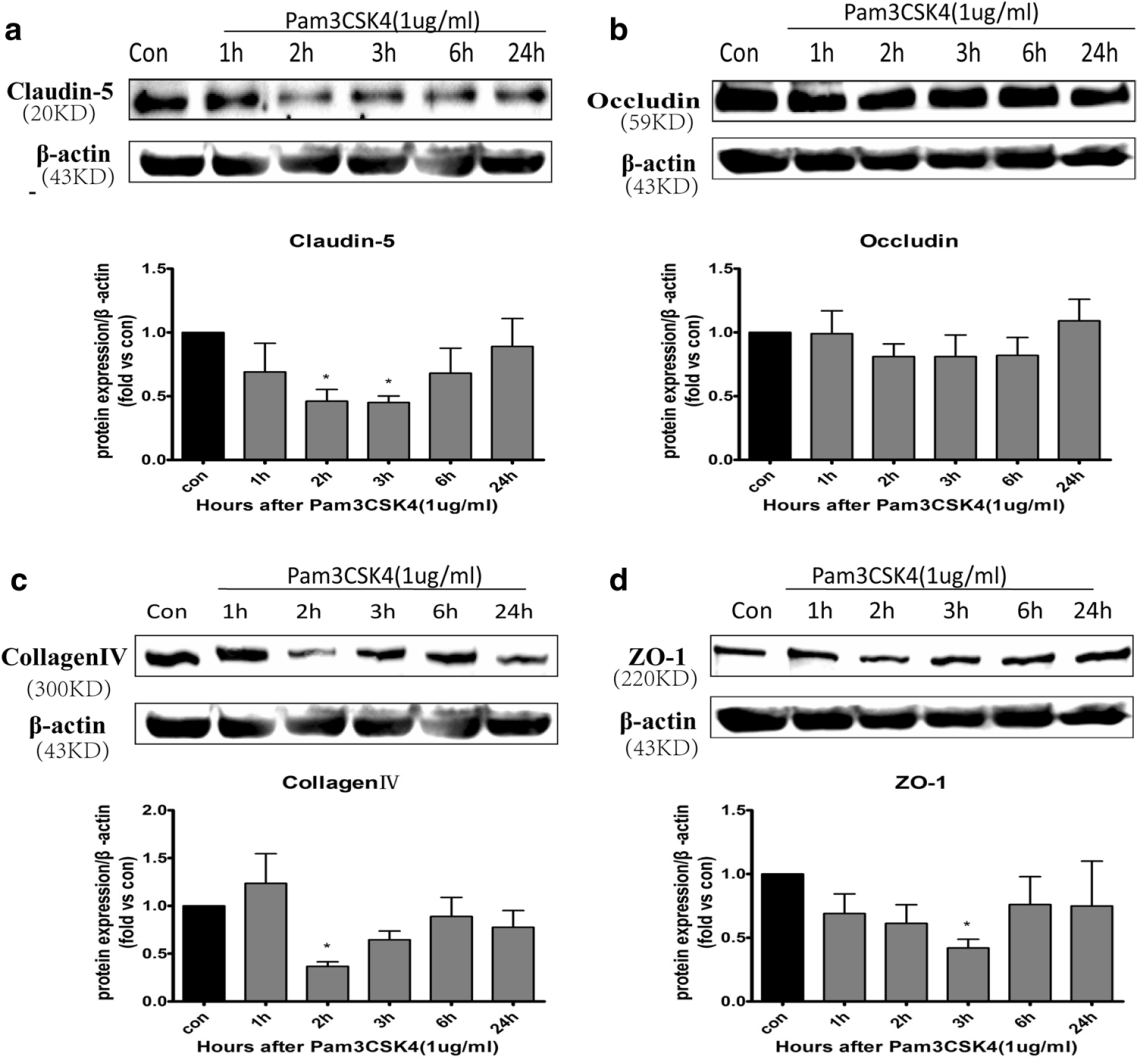
The protein expression levels of TJs in BMECs stimulated with Pam3CSK4. BMECs were stimulated with Pam3CSK4 (1 µg/mL) for 1, 2, 3, 6, and 24 h. Cells were collected for detection of claudin 5 (**a**), occludin (**b**), collagen IV (**c**), and ZO-1 (**d**) protein expression by western blot, and protein levels were quantified by ImageJ software and normalized with β-actin protein levels.*p < 0.05, **p < 0.001 as compared with control group, in which cells were treated with PBS

MMPs degrade TJ proteins (e.g., claudin 5 and occludin) and basal lamina proteins (e.g., laminin and collagen) in BMECs, leading to the disruption of the BBB [13, 14]. To observe whether TLR2 stimulation can destroy the TJs in BMECs, claudin 5, occludin, ZO-1, and collagen IV protein levels were measured by western blot after adding TLR2 agonist Pam3CSK4. It was found that levels of claudin 5 (Fig. 2a) and collagen IV (Fig. 2c) decreased at 2 h, and levels of ZO-1 (Fig. 2d) decreased at 3 h in BMECs after treatment with Pam3CSK4 (all *p* < 0.05). However, occludin levels did not change (Fig. 2b).

### Pam3CSK4 Induced the Phosphorylation of MAPK and NF-κB in BMECs

**Fig. 3:**
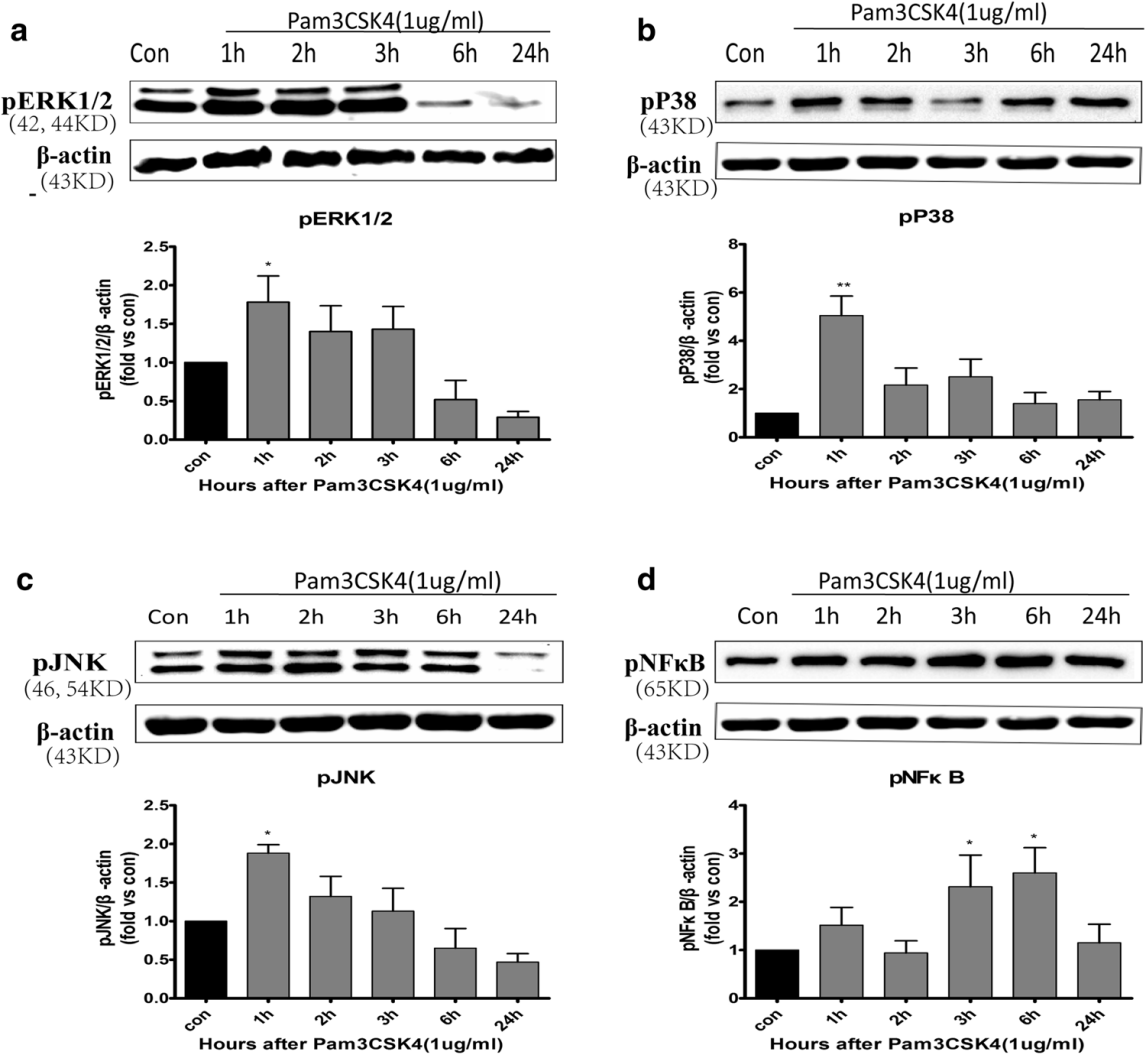
Pam3CSK4 induced ERK, JNK, p38, and NF-κB phosphorylation in BMECs. After the cells were treated with 1 µg/mL of Pam3CSK4 for different time periods, the expression of phosphorylated ERK (**a**), p38 (**b**), JNK (**c**), and NF-κB (**d**) was analyzed by western blot and normalized with β-actin protein levels. *p < 0.05, **p < 0.001 as compared with control group, in which cells were treated with PBS

After treating BMECs with 1 μg/mL Pam3CSK4 for 1, 2, 3, 6, and 24 h, we detected MAPK and NF-κB phosphorylation by western blotting. The phosphorylation of ERK1/2 (Fig. 3a), JNK (Fig. 3c), and p38 (Fig. 3b) MAPK was detected at 1 h (*p* < 0.05). The phosphorylation of ERK1/2 and JNK tended to remain for up to 3 h, though there was no statistical significance at 2 and 3 h (*p* > 0.05, Fig. 3a, c). NF-κB phosphorylation was induced at 3 and 6 h (*p* < 0.05, Fig. 3d).

### Pam3CSK4 Regulated the Expression of MMP-2/9 via ERK1/2, JNK, and NF-κB pathways

**Fig. 4:**
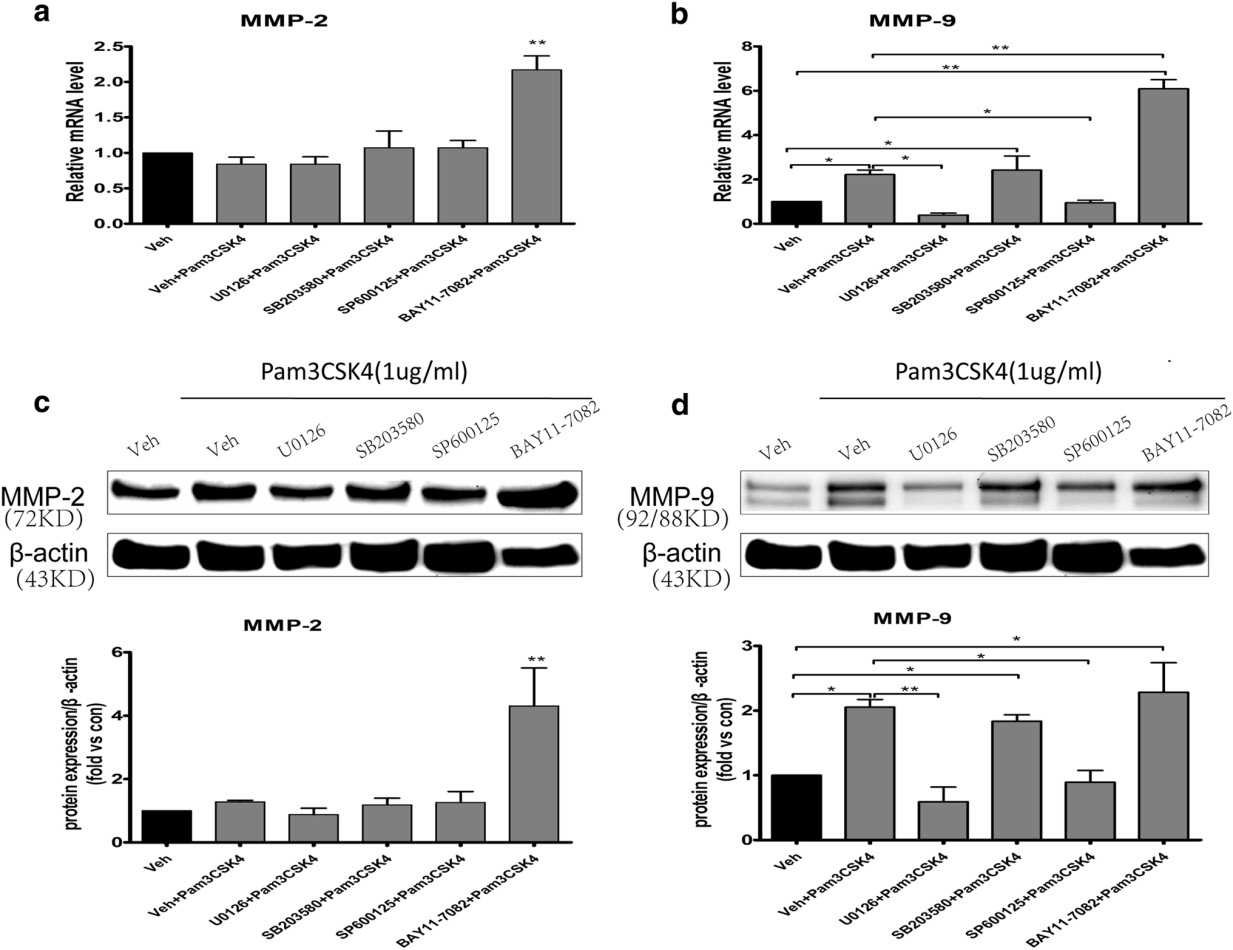
Pam3CSK4 influenced the expression levels of MMP-2 and MMP-9 via MAPK/NF-κB signaling pathways in BMECs. BMECs were pretreated with ERK1/2 inhibitor (U0126), p38 MAPK inhibitor (SB203580), JNK inhibitor (SP600125), and NF-κB inhibitor (BAY11-7082) or DMSO for 1 h and then treated with 1 µg/mL Pam3CSK4 for 6 h. The mRNA expression levels of MMP-2 (**a**), MMP-9 (**b**), and β-actin were analyzed by qRT-PCR. Cells were collected for detection of MMP-2 (**c**) and MMP-9 (**d**) protein expression by western blot, and protein levels were quantified by ImageJ software and normalized with β-actin protein levels. *p < 0.05, **p < 0.001 as compared with vehicle control group, in which cells were treated with PBS

To investigate the signaling pathways by which TLR2 regulates MMP-2/9 expression in BMECs, cells were pretreated with ERK1/2 inhibitor (20 µM U0126), p38 MAPK inhibitor (20 µM SB203580), JNK inhibitor (20 µM SP600125), and NF-κB inhibitor (10 µM BAY11-7082) or DMSO (0.1% vehicle control) for 1 h and then treated with 1 µg/mL Pam3CSK4 for 6 h. The expression of MMP-2 and MMP-9 was detected by qRT-PCR and western blotting. As shown in Fig. 4b, d, U0126 and SP600125 significantly inhibited MMP-9 expression, which was upregulated by Pam3CSK4 (all *p* < 0.05). Compared with that in the vehicle + Pam3CSK4 group, the upregulation of MMP-9 was not suppressed by SB203580 and BAY11-7082 (Fig. 4b, d). However, after pretreating cells with BAY11-7082, the mRNA and protein expression of MMP-2 (*p* < 0.001, Fig. 4a, c) and the mRNA of MMP-9 increased compared with that in the vehicle control and vehicle + Pam3CSK4 groups, respectively (*p* < 0.001, Fig. 4b).

## Discussion

There are three principal barriers between the blood and brain in CNS: the BBB, the blood–cerebral spinal fluid (CSF) barrier, and the arachnoid barrier [15]. BBB dysfunction is correlated with the pathophysiology of several neurological disorders [15], such as stroke, multiple sclerosis (MS), and Alzheimer’s disease (AD). BBB breakdown is also associated with cytokines, chemokines, and other substances, including MMPs [16].

The MMPs family includes more than 20 proteolytic enzymes [17]. They are commonly classified into four categories: collagenases, gelatinases (MMP-2 and MMP-9), stromelysins, matrilysins, and membrane-type MMPs [18]. The MMPs family is involved in tissue remodeling, cancer metastasis, chronic inflammation, and neurological disorders [19]. MMPs are also able to degrade several proteinases, growth factors, cell surface receptors, and cell–cell adhesion molecules [20]. In injured brain tissues, various cells express MMPs, including resident cells (endothelial cells, astrocytes, and neurons) and infiltrating inflammatory cells [9]. MMP expression is normally very low in the adult brain, but many studies have shown that several MMPs are activated and their levels increase after ischemic stroke [21, 22]. MMP activity is stringently modulated at four different levels: gene expression at the transcription level; compartmentalization; pro-enzyme activation; and inhibition of proteolysis [23].

When an agonist ligand binds to a TLR, adapter molecules will activate TLR signaling pathways. There are two types of TLR signaling pathways: myeloid differentiation factor 88 (MyD88)-dependent pathways and TIR-domain-containing adapter-inducing interferon-β (TRIF)-dependent (MyD88-independent) pathways [2]. TLR2 activates MAPKs [3] and transcription factors (NF-κB) through a MyD88-dependent signaling pathway, leading to the expression of proinflammatory cytokines such as interleukin(IL)-1, IL-6, and tumor necrosis factor (TNF)-α as well as MMP production. MAPKs include three major members: the extracellular signal-related kinases (ERKs), the c-Jun N-terminal kinases (JNKs)/stress-activated protein kinases, and p38 [24].

Recent studies have shown that MMP-2/9 are highly involved in CNS disorders. Previous researchers have revealed that MMP-9 expression is regulated by MAPKs in different cell types [25–27]. Additionally, transcription factor NF-κB participates in regulating the expression of MMPs in several cell types. Several TLR agonists can activate NF-κB and modulate MMP expression [26]. However, the molecular mechanism has not been clarified in BMECs of the BBB. Our study showed that TLR2 ligand Pam3CSK4 can upregulate MMP-9 expression significantly and downregulate MMP-2 in BMECs. Meanwhile, the stimulation of TLR2 led to downregulation of claudin-5, collagen IV, and ZO-1, which coincides with a study of the human cerebral endothelial cell line hCMEC/d3 [3]. MMP-2/9 are key mediators of inflammatory reactions, which possibly contribute to TJ degradation in BMECs and lack of BBB integrity in CNS disorders. A previous study showed that MMP-9 can damage TJ proteins [28]. It was reported that active MMP-9 induced the decrease of ZO-1 expression [29] and degradation of ZO-1 was attenuated in MMP-9 knock-out mice after ischemia [30]. It is reasonable to conclude that TLR2 activiation may degrade ZO-1 by increasing MMP-9 protein levels in BMECs. However, the time for MMP-9 protein induction is 3–6 h whereas the decrease in claudin-5 or collagen IV is observed only after 2 h treatment in this study. We only measured the amount of MMP-9 in cells by western blot and qRT-PCR. Therefore, it is speculated that the secreted MMP-9 in supernatant or other substances lead to the decrease of claudin-5 and collagen-IV after 2 h treatment.

Pam3CSK4 is the specific ligand of TLR2 [31] In this study, Pam3CSK4 induced the phosphorylation of ERK, JNK, and p38 MAPK in BMECs at 1 h and NF-κB phosphorylation at 3 and 6 h. Furthermore, to investigate the signaling pathways by which Pam3CSK4 affects the expression of MMP-2/9, we pretreated BMECs with inhibitors for 1 h and then treated cells with Pam3CSK4 for 6 h. ERK1/2 inhibitor (U0126) and JNK inhibitor (SP600125) significantly blocked MMP-9 expression induced by Pam3CSK4 at 6 h. Compared with that in the vehicle control, the upregulation of MMP-9 was not inhibited by p38 MAPK inhibitor (SB203580). It is concluded that TLR2 may regulate MMP-9 expression by ERK1/2 and JNK signaling pathways in BMECs. Conversely, after pretreating cells with NF-κB inhibitor (BAY11-7082), the mRNA and protein expression of MMP-2/9 obviously increased compared with that in the vehicle control, but only the mRNA expression of MMP-9 was significantly increased compared with that in the vehicle + Pam3CSK4 group. The results indicated that TLR2 negatively regulates the expression of MMP-2 and MMP-9 through the NF-κB signaling pathway in BMECs. A previous study demonstrated that TLR2 activated two downstream pathways, including the IKK complex and MAPK family, and then activated NF-κB and activator protein-1 (AP-1), resulting in expression of proinflammatory cytokines [32]. However, a study reported that phosphoinositide 3-kinase (PI3K) negatively regulated TLR2 signaling [33]. Therefore, it is inferred that regulation of MMP-2 expression by Pam3CSK4 involved a balance between the NF-κB signaling pathway and other signaling pathways, such as PI3K pathways. However, this experiment has a limitation. It was inappropriate to select Pam3CSK4 to stimulate BMECs for 6 h to investigate the signaling pathways by which TLR2 regulated MMP-2 expression, because there was no change in mRNA and protein levels of MMP-2 after Pam3CSK4 stimulation for 6 h in BMECs (Fig. 1a, b).

In conclusion, TLR2 regulated the expression of MMP-9 through ERK1/2 and JNK signaling pathways and negatively regulated the expression of MMP-2/-9 through the NF-κB signaling pathway in BMECs. The finding may provide novel insight into the molecular mechanism of MMP-2/-9 expression in BMECs.
